# Serum phosphate and 28-day mortality in adult sepsis with E.Coli infection: A critical care database study

**DOI:** 10.1371/journal.pone.0321063

**Published:** 2025-04-24

**Authors:** Ju Luo, Shifang Zhou, Ning Ding

**Affiliations:** 1 Department of Geriatrics, The Affiliated Changsha Central Hospital, Hengyang Medical School, University of South China, Changsha, China; 2 Department of Emergency Medicine, The Affiliated Changsha Central Hospital, Hengyang Medical School, University of South China, Changsha, China; Oregon State University, UNITED STATES OF AMERICA

## Abstract

**Objective:**

In this study, we aimed to explore the relationship between serum phosphate and clinical outcomes in sepsis with E.Coli infection based on a public database in order to help physicians do individualized medical decisions.

**Methods:**

We performed this retrospective study based on the Medical Information Mart for Intensive Care IV(MIMIC-IV) database (https://mimic.mit.edu/iv/). All the patients were hospitalized and serum phosphate was measured in 24 hours after hospitalization. E.Coli infection was confirmed by the positive blood culture of E.Coli in the database. Three models were utilized to investigate the relationship between serum phosphate and mortality in sepsis as follows: crude model (adjusted for none), model I (adjusted for age and gender) and model II (adjusted for all potential confounders). The smooth fitting curve was performed by the generalized additive model.

**Results:**

421 adult sepsis patients with E.Coli infection were included. The 28-day mortality was 10.69%(n=45). The median age was 70 and the proportion of males was 47.51%(n=200). The smooth fitting curve showed that the relationship between serum phosphate and 28-day mortality in sepsis with E.Coli infection was positive. When serum phosphate >2.1mg/dl, the relationship was significantly positive (OR=1.55, 95%CI:1.01–2.36, P=0.043).

**Conclusion:**

The positive relationship between serum phosphate and 28-day mortality in adult sepsis patients with E.Coli infection was found based on MIMIC-IV database.

## 1. Introduction

Escherichia coli (E. Coli), as a common gram-negative bacterium in the gastrointestinal system, has been an increasing cause for bloodstream infection and sepsis in recent years [1–4]. One recent study in United Kingdom revealed that E. Coli infections were found to be accounted for 75.65% of 1113 patients with laboratory-confirmed bloodstream infection [5]. In China, a 10-year retrospective research from 2010 to 2019 in a large tertiary hospital showed that in a total of 18,180 strains which were isolated from blood cultures, the most common was the E. coli (21.7%) [6]. Another study with 3819 patients undergoing cesarean section demonstrated that the incidence of sepsis was 0.84% and E. Coli was also the most common (46.15%) in sepsis patients [7]. One systemic review enrolled the literature from January 2007 to March 2018 and concluded that E. Coli was the main pathogen for 27% of documented bacteremia attacks [8].

Phosphate has been found to be related with various physiological functions [9,10]. It is crucial for cell metabolism, which puts an impact on contractility of muscle, transmission of neuron and transportation of electrocytes [11–13]. Phosphate, as an important element in human body, plays a vital role in almost all physiological activities [14]. Disturbance of serum phosphate was associated with adverse clinical outcomes in various diseases including cardiovascular diseases [15,16], malignant tumors [17] and infection [18].

However, the serum phosphate and prognosis in adult sepsis patients with E.Coli infection has not been investigated. In our study, we aimed to evaluate the association between serum phosphate and mortality in adult sepsis patients with E.Coli infection based on a public database in order to help physicians do individualized medical decisions.

## 2. Methods

### 2.1. Database, study cohort and definition

We performed this retrospective study based on the Medical Information Mart for Intensive Care IV(MIMIC-IV) database (https://mimic.mit.edu/iv/) [19]. The diagnosis of sepsis was based on the definition of Sepsis 3.0 [20]. E.Coli infection was confirmed by the positive blood culture of E.coli in the database. Sepsis patients with E.Coli infection were included in this study (n=535). Exclusion criteria were as follows:(1) missing data of serum phosphate within 24 hours after admission(n=1); (2) missing data >5% individual variables(n=113); (3) less than 18 years old (n=0).

### 2.2. Ethics approval and consent to participate

This study was conducted in accordance with Good Clinical Practice (Declaration of Helsinki 2002). MIMIC-IV was an anonymized public database. To apply for access to the database, we passed the Protecting Human Research Participants exam (No.32900964). The project was approved by the institutional review boards of the Massachusetts Institute of Technology (MIT) and Beth Israel Deaconess Medical Center (BIDMC) and was given a waiver of informed consent.

### 2.3. Data extraction and variables

Data in MIMIC-IV were extracted by PostgreSQL 9.6 software. Variables including age, gender, comorbidities (hypertension, renal disease, diabetes and coronary artery disease (CAD)), respiratory rate (RR), systolic blood pressure (SBP), diastolic blood pressure (DBP), heart rate (HR), phosphate, aspartate aminotransferase (AST), alanine aminotransferase(ALT), total bilirubin, total calcium, chloride, creatinine, glucose, international normalized ratio(INR), potassium, hemoglobin, platelet (PLT), prothrombin time (PT), partial thrombin time (PTT), urea nitrogen, sodium, white blood cells (WBC), scores of sequential organ failure assessment (SOFA) and acute physiology and chronic health evaluation (APACHEII), days of length of stay (LOS) in ICU and hospital, and 28-day mortality were extracted. Only the first value of each variable within 24 hours after admission was enrolled for analysis.

### 2.4. Statistical analysis

Firstly, based on tertiles of serum phosphate, sepsis patients with E.Coli infection were divided into three groups. Variables were showed as follows: 1) continuous variables as medians; 2) categories variables as percentages or frequencies. Mann–Whitney U-test or Chi-squared test were used for analyzing the variables between different three groups. Secondly, univariable and multivariable analyses were applied to explore the associations between different variables and 28-day mortality in sepsis with E.Coli infection. Thirdly, three models were utilized to investigate the relationship between serum phosphate and 28-mortality in sepsis as follows: crude model (adjusted for none), model I (adjusted for age and gender) and model II (adjusted for age; gender; HR; SBP; DBP; RR; ALT, AST, total bilirubin; total calcium; chloride; creatinine; glucose; hemoglobin; INR; PLT; PT; PTT; potassium; sodium; urea nitrogen; WBC; renal disease; CAD; diabetes; hypertension; APAHCEII; SOFA). Fourthly, a smooth fitting curve for indicating the relationship between serum phosphate and 28-day mortality in sepsis with E.Coli infection was performed by the generalized additive model. Two models including linear model and non-linear model were compared. If the P value <0.05, the non-linear model was selected. If not, the linear model was better. Finally, the subgroups analyses were done for evaluating the stability of results in the subgroups.

The statistical software packages R (http://www.R-project.org) and EmpowerStats (http://www.empowerstats.com) were applied to complete this study. Statistical significance was defined when the P-value <0.05.

## 3. Results

### 3.1. General characteristics of the study cohort

In the present study, 421 sepsis with E.Coli infection were finally included (S1 Fig). Based on tertiles of serum phosphate, sepsis patients with E.Coli infection were divided into three groups. (Q1:<2.7mg/dl(n=130), Q2: 2.7–3.5mg/dl(n=128), Q3:>3.5 mg/dl(n=163)) (**Table 1**). The 28-day mortality was 10.69%(n=45). The median age was 70 and the proportion of males was 47.51%(n=200). Based on tertiles of serum phosphate, variables in three groups showed significant differences including renal disease (P=0.021), HR(P=0.027), creatinine (P<0.001), chloride(P=0.007), INR(P=0.035), hemoglobin(P=0.026), PLT (P=0.005), potassium(P<0.001), PT(P=0.029), urea nitrogen(P<0.001), APCHEII score (P=0.007) and SOFA score(P=0.026). In Q3 group, the days of LOS in ICU and hospital were significantly longer (both P=0.005). The 28-day mortalities in Q1-Q3 groups were 4.62%, 7.81% and 17.79%, respectively(P<0.001).

**Table 1:** General characteristics of the sepsis patients with E.Coli infection.

Serum phosphate(mg/dl)(tertiles)					
Variables	Total	Q1(<2.7)	Q2(2.7–3.5)	Q3(>3.5)	P-value
Number	421	130	128	163	
Age(years)	70.00 (60.00-81.00)	67.00 (58.00-81.00)	72.00 (60.75-81.00)	70.00 (61.00-81.00)	0.421
**Gender(n,%)**					0.750
Male	200 (47.51%)	65 (50.00%)	58 (45.31%)	77 (47.24%)	
Female	221 (52.49%)	65 (50.00%)	70 (54.69%)	86 (52.76%)	
**Comorbidities(n,%)**					
Hypertension	196 (46.56%)	68 (52.31%)	58 (45.31%)	70 (42.94%)	0.264
CAD	89 (21.14%)	25 (19.23%)	29 (22.66%)	35 (21.47%)	0.790
Diabetes	24 (5.70%)	7 (5.38%)	7 (5.47%)	10 (6.13%)	0.954
Renal disease	17 (4.04%)	2 (1.54%)	3 (2.34%)	12 (7.36%)	0.021
**Variables**					
HR(beats/min)	98.00 (83.00-110.00)	101.00 (87.00-113.00)	93.00 (81.00-107.00)	99.00 (83.00-112.00)	0.027
RR(beats/min)	20.00 (17.00-25.00)	21.00 (17.00-25.00)	21.00 (18.00-24.00)	20.00 (16.00-24.00)	0.278
SBP(mmHg)	108.00 (95.00-125.00)	107.50 (93.25-120.75)	108.50 (96.00-127.25)	109.00 (96.00-126.50)	0.358
DBP(mmHg)	60.00 (51.00-71.00)	59.00 (50.00-70.00)	61.00 (53.00-71.25)	60.50 (50.00-72.00)	0.445
Phosphate(mg/dL)	3.20 (2.40-4.00)	2.10 (1.72-2.40)	3.10 (2.80-3.30)	4.30 (3.80-5.00)	<0.001
ALT(IU/L)	40.00 (19.00-101.00)	39.00 (17.75-102.50)	41.00 (20.75-102.75)	38.50 (18.25-75.75)	0.571
AST(IU/L)	54.00 (29.00-118.00)	54.50 (27.00-119.75)	62.00 (29.75-133.00)	51.50 (31.00-104.50)	0.733
Total bilirubin(mg/dL)	0.90 (0.50-2.30)	0.85 (0.40-2.12)	0.90 (0.47-2.10)	1.00 (0.50-2.50)	0.476
Total calcium(mg/dL)	7.90 (7.30-8.50)	7.70 (7.20-8.30)	8.00 (7.40-8.50)	7.90 (7.30-8.50)	0.071
Creatinine(mg/dL)	1.30 (1.00-1.90)	1.10 (0.90-1.58)	1.25 (0.90-1.70)	1.60 (1.10-2.45)	<0.001
Chloride(mmol/L)	105.00 (101.00-109.00)	106.00 (103.00-111.00)	105.00 (100.50-109.00)	104.00 (100.00-108.00)	0.007
Glucose(mg/dL)	121.00 (98.00-163.00)	129.00 (101.50-170.50)	117.00 (94.50-145.50)	120.00 (98.00-163.50)	0.177
INR	1.40 (1.20-1.70)	1.30 (1.20-1.50)	1.40 (1.20-1.70)	1.40 (1.22-2.00)	0.035
Hemoglobin(g/dL)	10.50 (9.10-12.00)	11.05 (9.50-12.28)	10.60 (9.35-12.00)	10.20 (8.85-11.55)	0.026
PLT(*10^9^/L)	161.50 (115.00-236.25)	143.00 (101.00-205.25)	166.00 (120.00-229.00)	175.00 (123.50-258.00)	0.005
Potassium(mmol/L)	4.00 (3.60-4.40)	3.70 (3.40-4.10)	3.95 (3.60-4.30)	4.20 (3.90-4.60)	<0.001
PT(s)	15.10 (13.70-18.60)	14.80 (13.62-16.50)	14.90 (13.70-18.35)	15.50 (13.80-21.45)	0.029
PTT(s)	34.40 (29.78-40.80)	32.65 (29.10-39.48)	34.50 (30.75-40.08)	35.75 (29.87-44.08)	0.083
Urea nitrogen(mg/dL)	27.00 (18.00-40.00)	20.50 (15.25-30.75)	27.00 (21.00-38.25)	33.00 (21.00-51.00)	<0.001
WBC(*10^9^/L)	12.60 (7.75-19.20)	12.15 (6.88-17.65)	12.90 (8.65-19.75)	12.60 (7.55-19.10)	0.274
Sodium(mmol/L)	138.00 (135.00-141.00)	139.00 (135.00-141.00)	137.00 (134.00-141.00)	138.00 (135.00-141.00)	0.252
APACHEII	12.00 (9.00-14.00)	11.00 (9.00-14.00)	11.00 (9.00-14.00)	13.00 (10.00-15.00)	0.007
SOFA	3.00 (2.00-4.00)	3.00 (2.00-4.00)	3.00 (2.00-4.00)	3.00 (2.00-5.00)	0.026
**Clinical outcomes(days)**					
LOS in ICU	2.88 (1.62-5.91)	2.62 (1.73-4.49)	2.40 (1.46-4.96)	3.83 (1.91-7.40)	0.005
LOS in hospital	7.86 (5.41-13.59)	6.76 (5.31-10.68)	7.76 (5.33-11.83)	9.70 (5.72-15.82)	0.005
**28-day mortality(n,%)**	45 (10.69%)	6 (4.62%)	10 (7.81%)	29 (17.79%)	<0.001

Abbreviations: E.Coli= Escherichia coli, SBP=systolic blood pressure, DBP= diastolic blood pressure, HR= heart rate, RR=respiratory rate, ALT=alanine aminotransferase, AST= aspartate aminotransferase, INR= international normalized ratio, WBC=white blood cells, PLT=platelet, PT= prothrombin time, PTT=partial thrombin time, CAD= coronary artery disease, APACHE=acute physiology and chronic health evaluation, SOFA=sequential organ failure assessment; LOS=length of stay, ICU=intensive care unit.

### 3.2. Univariable and multivariable analysis for 28-day mortality in sepsis with E.Coli infection

In **Table 2**, univariable and multivariable analysis for 28-day mortality in sepsis with E.Coli infection were demonstrated. Multivariable analysis showed that variables including renal disease(P=0.023), HR(P=0.016), phosphate(P=0.003), total calcium (P=0.003) were significantly associated with 28-day mortality in sepsis with E.Coli infection.

**Table 2:** Univariable and multivariable analysis for 28-day mortality in sepsis.

Variables	Univariable (OR,95%CI, P)	Multivariable (OR,95%CI, P)
Age(years)	1.01 (0.99, 1.04) 0.201	1.01 (0.98, 1.05) 0.467
Gender		
Male	Ref.	Ref.
Female	0.70 (0.37, 1.30) 0.254	0.39 (0.15, 1.01) 0.053
Hypertension		
No	Ref.	Ref.
Yes	0.74 (0.40, 1.39) 0.352	0.76 (0.30, 1.92) 0.562
Renal disease		
No	Ref.	Ref.
Yes	2.72 (0.85, 8.74) 0.092	13.50 (1.43, 127.67) 0.023
CAD		
No	Ref.	Ref.
Yes	0.79 (0.35, 1.76) 0.559	0.69 (0.23, 2.04) 0.498
Diabetes		
No	Ref.	Ref.
Yes	0.35 (0.05, 2.65) 0.308	0.85 (0.09, 7.97) 0.887
HR(beats/min)	1.01 (1.00, 1.03) 0.086	1.04 (1.01, 1.07) 0.016
RR(beats/min)	1.03 (0.98, 1.08) 0.318	0.92 (0.85, 1.00) 0.059
SBP(mmHg)	1.00 (0.99, 1.01) 0.973	1.00 (0.98, 1.02) 0.959
DBP(mmHg)	1.00 (0.98, 1.01) 0.789	0.98 (0.95, 1.01) 0.255
Phosphate(mg/dL)	1.42 (1.17, 1.73) <0.001	1.79 (1.22, 2.62) 0.003
ALT(IU/L)	1.00 (1.00, 1.00) 0.641	1.00 (1.00, 1.01) 0.164
AST(IU/L)	1.00 (1.00, 1.00) 0.632	1.00 (1.00, 1.00) 0.269
Total bilirubin(mg/dL)	1.02 (0.93, 1.11) 0.717	0.97 (0.86, 1.09) 0.597
Total calcium(mg/dL)	1.29 (0.98, 1.70) 0.068	2.27 (1.33, 3.86) 0.003
Creatinine(mg/dL)	1.11 (0.94, 1.31) 0.226	0.65 (0.40, 1.07) 0.094
Chloride(mmol/L)	0.99 (0.95, 1.04) 0.707	1.06 (0.95, 1.19) 0.292
Glucose(mg/dL)	1.00 (0.99, 1.00) 0.494	1.00 (0.99, 1.00) 0.181
INR	0.92 (0.66, 1.28) 0.619	0.78 (0.02, 26.16) 0.889
Hemoglobin(g/dL)	0.97 (0.83, 1.13) 0.708	1.00 (0.79, 1.28) 0.983
PLT(*10^9^/L)	1.00 (1.00, 1.00) 0.896	1.00 (0.99, 1.00) 0.671
Potassium(mmol/L)	1.40 (0.99, 1.97) 0.055	0.91 (0.51, 1.64) 0.762
PT(s)	0.99 (0.96, 1.02) 0.572	1.02 (0.70, 1.47) 0.924
PTT(s)	1.00 (0.99, 1.02) 0.801	1.00 (0.97, 1.02) 0.773
Urea nitrogen(mg/dL)	1.01 (1.00, 1.02) 0.195	1.02 (0.99, 1.04) 0.211
WBC(*10^9^/L)	1.00 (0.98, 1.03) 0.699	1.02 (0.99, 1.05) 0.169
Sodium(mmol/L)	1.01 (0.96, 1.06) 0.754	0.96 (0.84, 1.09) 0.507
APACHEII	1.11 (1.03, 1.19) 0.008	1.07 (0.93, 1.23) 0.325
SOFA	1.18 (1.02, 1.38) 0.029	1.22 (0.85, 1.76) 0.289

Abbreviations: SBP=systolic blood pressure, DBP= diastolic blood pressure, HR= heart rate, RR=respiratory rate, ALT=alanine aminotransferase, AST= aspartate aminotransferase, INR= international normalized ratio, WBC=white blood cells, PLT=platelet, PT= prothrombin time, PTT=partial thrombin time, CAD= coronary artery disease, APACHE=acute physiology and chronic health evaluation, SOFA=sequential organ failure assessment; OR=odds ratio, CI= confidential interval.

### 3.3. Association between serum phosphate and 28-day mortality in sepsis with E.Coli infection

In **Table 3**, three models were constructed for exploring the association between serum phosphate and 28-day mortality. In crude model (adjusted for none), with each 1mg/dl increment in serum phosphate, the risk of 28-day mortality increased by 42% (OR=1.42, 95%CI: 1.17–1.73, P<0.001). In model I (adjusted for age and gender), the risk of 28-day mortality increased by 46% (OR=1.46, 95%CI: 1.19–1.78, P<0.001). In model II (adjusted for age; gender; HR; SBP; DBP; RR; ALT, AST, total bilirubin; total calcium; chloride; creatinine; glucose; hemoglobin; INR; PLT; PT; PTT; potassium; sodium; urea nitrogen; WBC; renal disease; CAD; diabetes; hypertension; APAHCEII; SOFA), with each 1mg/dl increment in serum phosphate, the risk of 28-day mortality increased by 79% (OR=1.79, 95%CI: 1.22–2.62, P=0.003).

**Table 3:** Association between serum phosphate and 28-day mortality in three models.

OR (95%CI), P value			
Exposure	Crude model	Model I	Model II
Serum phosphate (per 1mg/dl increment)	1.42 (1.17, 1.73) <0.001	1.46 (1.19, 1.78) <0.001	1.79 (1.22, 2.62) 0.003
Serum phosphate(mg/dl) tertiles			
Q1	Ref.	Ref.	Ref.
Q2	1.75 (0.62, 4.97) 0.292	1.74 (0.61, 4.94) 0.302	1.74 (0.44, 6.91) 0.433
Q3	4.47 (1.80, 11.14) 0.001	4.46 (1.79, 11.14) 0.001	6.26 (1.72, 22.81) 0.005
P for trend	<0.001	<0.001	0.004

Crude model adjusted for: None;

Model I adjusted for: age; gender;

Model II adjusted for: age; gender; HR; SBP; DBP; RR; ALT, AST, total bilirubin; total calcium; chloride; creatinine; glucose; hemoglobin; INR; PLT; PT; PTT; potassium; sodium; urea nitrogen; WBC; renal disease; CAD; diabetes; hypertension; APAHCEII; SOFA.

Abbreviations: SBP=systolic blood pressure, DBP= diastolic blood pressure, HR= heart rate, RR=respiratory rate, ALT=alanine aminotransferase, AST= aspartate aminotransferase, INR= international normalized ratio, WBC=white blood cells, PLT=platelet, PT= prothrombin time, PTT=partial thrombin time, CAD= coronary artery disease, APACHE=acute physiology and chronic health evaluation, SOFA=sequential organ failure assessment; OR=odds ratio, CI= confidential interval.

Based on tertiles of serum phosphate, the associations between Q1-Q3 groups and 28-day mortality in three models were analyzed. Compared with Q1 group, the ORs of 28-day mortality in Q3 group were 4.47 (95%CI: 1.80–11.14, P=0.001, crude model), 4.46 (95%CI: 1.79–11.14, P=0.001, model I), and 6.26 (95%CI: 1.72–22.81, P=0.005, model II), respectively. With the serum phosphate increasing, the risk of mortality increased significantly and all P-value for trend in three models were less than 0.05 (P<0.001 in crude model, P<0.001 in model I, and P=0.004 in model II).

In **Table 4**, two models including the linear model and two-segment non-linear model were applied to fit the relationship between serum phosphate and mortality. In the two-segment non-linear model, the turning point of serum phosphate was 2.1mg/dl. When the serum phosphate >2.1mg/dl, the relationship between serum phosphate and 28-day mortality was significantly positive (P=0.043). While the serum phosphate ≤2.1mg/dl, the relationship was not significant (P=0.212).

**Table 4:** The results of the two-piecewise linear model.

	Number (%)	OR (95%CI), P-value
Model I: The linear model	421(100%)	1.79 (1.22, 2.62), 0.003
Model II: Two-segment non-linear model		
The turning point of serum phosphate(mg/dL)		2.1
≤2.1 (left side, slope 1)	69(16.39%)	94.83 (0.07, 120327.73), 0.212
>2.1 (right side, slope 2)	352(83.61%)	1.55 (1.01, 2.36), 0.043
Slope 2 to slope 1		0.02 (0.00, 23.07), 0.266
Predicted at		-2.67 (-3.29, -2.06)
P for the log-likelihood ratio test		0.106

Model adjusted for: age; gender; HR; SBP; DBP; RR; ALT, AST, total bilirubin; total calcium; chloride; creatinine; glucose; hemoglobin; INR; PLT; PT; PTT; potassium; sodium; urea nitrogen; WBC; renal disease; CAD; diabetes; hypertension; APAHCEII; SOFA.

Abbreviations: SBP=systolic blood pressure, DBP= diastolic blood pressure, HR= heart rate, RR=respiratory rate, ALT=alanine aminotransferase, AST= aspartate aminotransferase, INR= international normalized ratio, WBC=white blood cells, PLT=platelet, PT= prothrombin time, PTT=partial thrombin time, CAD= coronary artery disease, APACHE=acute physiology and chronic health evaluation, SOFA=sequential organ failure assessment; OR=odds ratio, CI= confidential interval.

**Fig 1:**
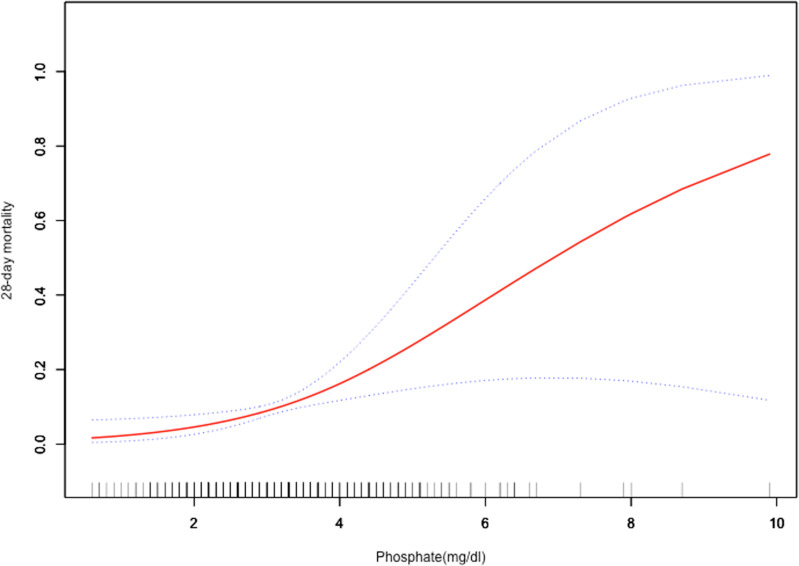
The smooth fitting curve was performed for indicating the relationship between serum phosphate and 28-day mortality in adult sepsis patients with E.Coli infection. Abbreviation: E.Coli= Escherichia coli.

In **Fig 1**, the smooth fitting curve was performed for indicating the relationship between serum phosphate and 28-day mortality in sepsis with E.Coli infection was positive.

### 3.4. Kaplan-Meier analysis for survival probability based on tertiles of serum phosphate

**Fig 2:**
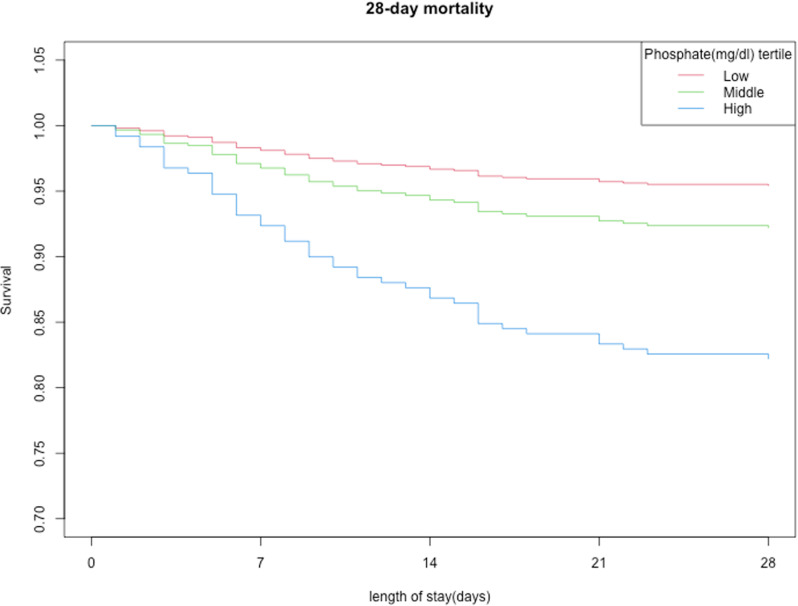
Kaplan-Meier analysis for survival probability based on tertiles of serum phosphate.

In **Fig 2**, Kaplan-Meier analysis for survival probability based on tertiles of serum phosphate showed that in the high level of serum phosphate group (Q3 group), the survival probability was significantly lowest(P<0.001).

### 3.5. Subgroup analysis

In S1 Table, subgroup analyses were investigated. The relationship between serum phosphate and mortality in different subgroups including gender, age, renal disease, CAD, diabetes, hypertension, WBC, hemoglobin, total bilirubin, urea nitrogen and creatinine were comparatively stable.

## 4. Discussion

In the study, the positive relationship between serum phosphate and clinical outcomes in adult sepsis patients with E.Coli infection was found based on MIMIC-IV database. As far as we know, this was the first study to investigate the association between serum phosphate and outcomes in adult sepsis patients with E.Coli infection in MIMIC-IV database.

Previous studies showed that serum phosphate was not only linked with various diseases, but also was an indicator for clinical outcomes. A cohort on the community based on older population revealed that disturbance of serum phosphate was associated with diabetes and metabolic syndrome [21]. In stroke patients, higher levels of serum phosphate were related with cerebral small vascular disorders [22]. One research from China demonstrated that in myocardial infarction patients, those with serum phosphate >4.5mg/dl had the highest risk of mortality (Hazard ratio(HR)=1.46 (95% CI: 1.35–1.83) compared to those with normal range of serum phosphate levels [23]. A large-scale retrospective study found that in emergency department, patients with lower serum phosphate levels had increased mortality risk of 30-day (OR=1.3, 95% CI:1.1–1.4) and 90-day (OR=1.2, 95%CI: 1.1–1.3) [24].

In our study, higher levels of serum phosphate were associated with increased risk of mortality in sepsis patients with E.Coli infection. First, sepsis leads to elevated levels of serum phosphate due to damage of cellular metabolism and the release of intracellular phosphate [25]. In addition, metabolic acidosis due to sepsis also causes the transcellular shifts of phosphate [26,27]. Second, increased level of serum phosphate disturbs the balance of phosphate and calcium. It makes vascular calcification and tubular cell damage, leading to organ dysfunction [28]. Moreover, current evidences clarified that higher levels of serum phosphate might enhance cell apoptosis, interfere cell migration and impair the function of endothelial cell, which may result in tissue ischemia and poor prognosis [29,30].

The main strength of this study was that the relationship between serum phosphate and prognosis in adult sepsis patients with E.Coli infection based on a large-scale public database was identified. However, several limitations should be considered. First, 28-day mortality was our mortality assessment and the mortality might be influenced by not only the sepsis but also other factors of mortality including exacerbation of underlying comorbidities. Hence, we couldn’t make a conclusion that the cause-effect between serum phosphate and mortality in sepsis with E.Coli infection was identified. Second, some potential confounders including clinical treatments (such as dialysis, renal replacement therapy) and socio-demographic parameters were not enrolled due to the lack of some data in the database. Third, serum phosphate might be affected by some factors including dietary intake and hormone levels of phosphate-regulating. In the further study, more variables would be enrolled for comprehensively analyzed.

## 5. Conclusion

The positive relationship between serum phosphate and 28-day mortality in adult sepsis patients with E.Coli infection was found based on MIMIC-IV database.

## Supporting information

S1 Fig Study design and patients enrollment. (ZIP)

S1 Table Subgroups analyses. (ZIP)
