# Supplementary material for: Serum phosphate and 28-day mortality in adult sepsis with E.Coli infection: A critical care database study
Source: PLoS One. 2025 Apr 24;20(4):e0321063. doi: 10.1371/journal.pone.0321063 (PMC12021143; doi:10.1371/journal.pone.0321063)
Supplement: S1 Fig — (ZIP) [file pone.0321063.s001.zip › μ£¬σæ╜σÉìμûçΣ╗╢σñ╣/S1Table.docx]

**S1 Table: Subgroups analyses**

| Variables | OR (95%CI) | P for interaction |
| --- | --- | --- |
| Gender |  | 0.816 |
| Male | 1.39 (1.07, 1.81) 0.013 |  |
| Female | 1.46 (1.09, 1.96) 0.011 |  |
| Age (tertiles) |  | 0.525 |
| Low | 1.63 (1.18, 2.24) 0.003 |  |
| Middle | 1.22 (0.83, 1.80) 0.314 |  |
| High | 1.42 (0.99, 2.04) 0.058 |  |
| Renal disease |  | 0.758 |
| No | 1.41 (1.15, 1.74) 0.001 |  |
| Yes | 1.26 (0.64, 2.49) 0.504 |  |
| CAD |  | 0.922 |
| No | 1.43 (1.16, 1.75) <0.001 |  |
| Yes | 1.38 (0.73, 2.61) 0.325 |  |
| Diabetes |  | 0.313 |
| No | 1.41 (1.15, 1.71) <0.001 |  |
| Yes | 5.64 (0.22, 142.96) 0.294 |  |
| Hypertension |  | 0.950 |
| No | 1.41 (1.11, 1.79) 0.005 |  |
| Yes | 1.43 (1.01, 2.02) 0.045 |  |
| WBC (tertiles) |  | 0.868 |
| Low | 1.42 (1.04, 1.95) 0.026 |  |
| Middle | 1.57 (1.08, 2.28) 0.018 |  |
| High | 1.37 (0.96, 1.96) 0.087 |  |
| Hemoglobin (tertiles) |  | 0.695 |
| Low | 1.31 (0.98, 1.75) 0.065 |  |
| Middle | 1.60 (1.07, 2.39) 0.022 |  |
| High | 1.51 (1.01, 2.28) 0.047 |  |
| Total bilirubin(tertiles) |  | 0.133 |
| Low | 2.16 (1.34, 3.48) 0.002 |  |
| Middle | 1.21 (0.85, 1.71) 0.289 |  |
| High | 1.45 (1.00, 2.10) 0.049 |  |
| Urea nitrogen (tertiles) |  | 0.443 |
| Low | 2.17 (1.08, 4.37) 0.029 |  |
| Middle | 1.40 (0.92, 2.14) 0.117 |  |
| High | 1.35 (1.03, 1.77) 0.032 |  |
| Creatinine (tertiles) |  | 0.867 |
| Low | 1.27 (0.81, 1.99) 0.296 |  |
| Middle | 1.55 (0.86, 2.78) 0.147 |  |
| High | 1.40 (1.07, 1.84) 0.014 |  |

**Abbreviations:** CAD=coronary artery disease, WBC=white blood cell; OR=odds ratio, CI= confidential interval.
