# Supplementary figures and images for: Serum phosphate and 28-day mortality in adult sepsis with E.Coli infection: A critical care database study
Source: PLoS One. 2025 Apr 24;20(4):e0321063. doi: 10.1371/journal.pone.0321063 (PMC12021143; doi:10.1371/journal.pone.0321063)

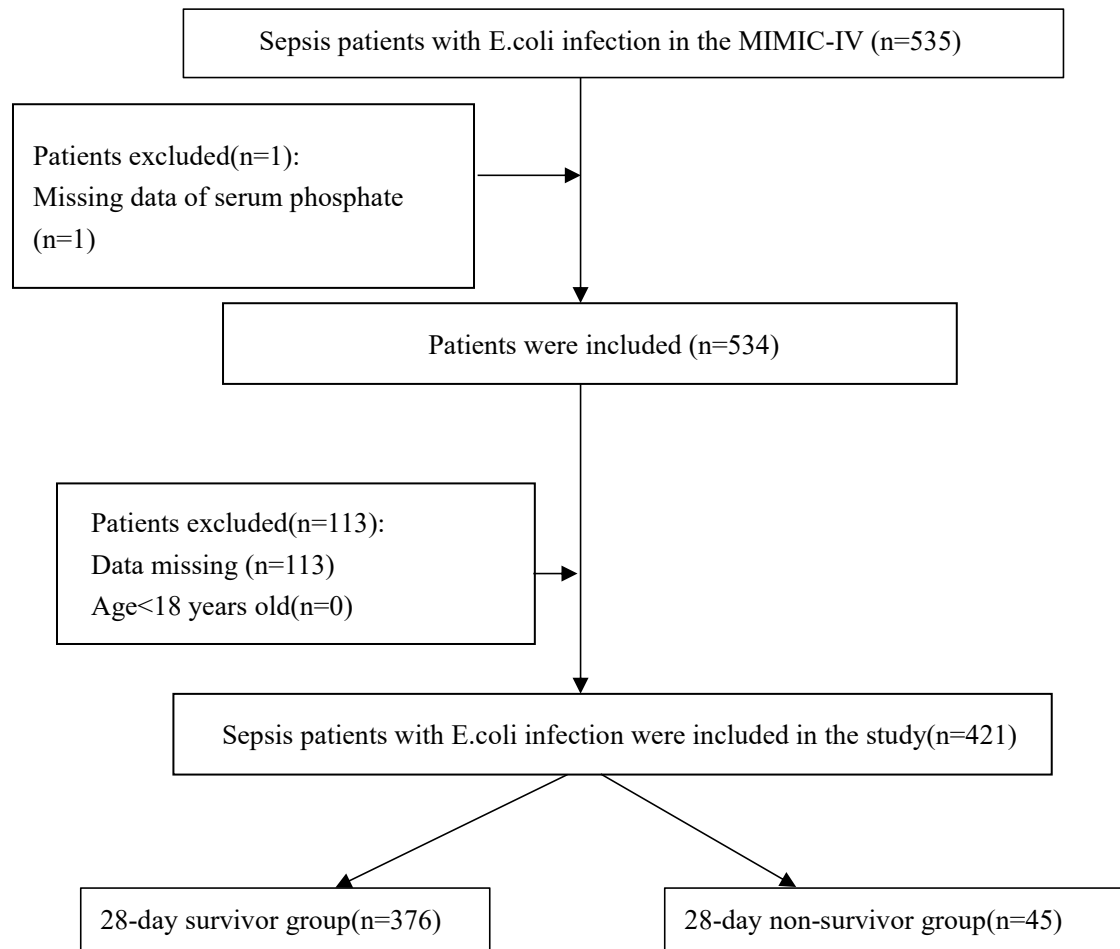

**S1 Figure : Study design and patients enrollment.**

**Abbreviation:** E.coli= Escherichia coli

Supplement: S1 Fig — (ZIP) [file pone.0321063.s001.zip › μ£¬σæ╜σÉìμûçΣ╗╢σñ╣/S1 Figure.pdf]
